# Experimental Study and Failure Criterion Analysis on Combined Compression-Shear Performance of Self-Compacting Concrete

**DOI:** 10.3390/ma13030713

**Published:** 2020-02-05

**Authors:** Jingrong Wang, Faxiang Xie, Chuanlong Zhang, Jing Ruan

**Affiliations:** 1 College of Mechanics and Materials, Hohai University, Nanjing 210098, China; wangjingrong@hhu.edu.cn; 2 College of Civil and Transportation Engineering, Hohai University, Nanjing 210098, China; zclhhu@hhu.edu.cn; 3 Jiangsu Provincial Transportation Construction Bureau, Nanjing 210004, China; tzbridge@163.com

**Keywords:** self-compacting concrete, compression-shear stress, failure criterion, mechanical performance, mechanical experiments, numerical regression

## Abstract

To investigate the combined compression-shear performance of self-compacting concrete (SCC), eight groups of concrete specimens under different axial compression ratios were designed, and the composite performance under different axial stresses was carried out by hydraulic servo machine. The uniaxial and tensile splitting strength of SCC were also included in the study. The failure modes of SCC were presented, discussed, and compared with normal concrete (NC). The characteristic points of stress-strain curves of SCC specimens from the experiments were extracted and analyzed under different axial compression stress. Based on the experimental results, the shear strength of compression-shear load was divided into cohesive stress and residual friction stress. The variation of residual stress and cohesive stress under the combined compression-shear stress was analyzed, and the relationship was obtained by numerical regression. Research results indicated that the residual stress increases linearly with the compression stress while the cohesive stress increased at first and then decreased. The research found that the friction coefficient of SCC was much smaller than NC due to the lack of interlocking effect. Utilizing the compression-shear strength of SCC, the material failure criteria of SCC were proposed from the view of shear failure strength and octahedral stress space, which could fit the experimental results confidently following the mathematical regression analysis. The comparison with data from other literature shows favorable consistence with the obtained criteria. The results of the study could be beneficial complement in engineering practices where SCC was applicable.

## 1. Introduction

Self-compacting concrete (SCC) is a kind of fresh concrete which has an ability to flow under its own weight, fill the required space or formwork completely, and produce a dense and adequately homogeneous material without a need for mechanical compaction [1]. In 1988, the concept of SCC was first proposed by Okumara et al. [2,3] in Japan, and then Ozawa et al. [4] conducted the research on the working performance of different mix proportions, and determined the method to obtain high fluidity with less aggregate content, lower water-powder ratio, and super plasticizer. In recent years, SCC has attracted extensive attention and application because it can achieve better filling and wrapping ability for steel bars, which also has better concrete appearance and can reduce labor demand. Scholars world widely have conducted comprehensive research on SCC, mainly including the mixture ratio, working performance, mechanical properties, and durability of SCC. H. Upadhyay et al. [5] summarized the test and design methods of SCC. Su et al. [6] proposed a new SCC mixture ratio design method, which is simpler than the method proposed by Japan instant concrete association and can meet the performance requirements of different test methods. Shi et al. [7] analyzed the mix proportion and basic performance of self-compacting lightweight aggregate concrete containing glass powder. In order to ensure the working performance of SCC, its testing methods include filling property, segregation resistance and gap passing property. In Chinese national code [8], the characteristic items, such as slump, expansion time, J-ring, and jumping table are used to evaluate the working performance of SCC. The British standard [9] adopts more different methods to test the performance of SCC, such as L-box, U-box, and GTM wet sieve stability. Holschmacher [10] created a database to analysis the performance of SCC with different mix proportions. Domone [11] collected more than 71 studies of the hardened mechanical properties of SCC. The properties, including uniaxial compressive strength, tensile strength, and the elastic modulus, as well as the bond of SCC to reinforcing steel bars were discussed. Khayat et al. [12] gave the comprehensive review of state-of-art mechanical properties of SCC. In the recent 20 years, SCC has been used and will have more potential usage in civil engineering, such as the retrofit of existing buildings [13] or used as main load-bearing elements in structures [14]. However, due to the lack of experimental data, the biaxial or multiaxial results were rarely explored and discussed.

The stress of concrete in structures are often complex. Concrete structures in actual projects are not only subjected to uniaxial stress, but often subjected to composite stresses [7,15,16]. In bridge engineering practices, the structural elements, such as deep beams, corbels, bearing padding stones, and other components where SCC has potential usages are subjected to typical compression-shear composite stress, so it is of great importance to study the performance of SCC in multi-stress states. However, reports on multi-axial stress performance of SCC, including composite compression-shear stress, are relatively bare. Yu et al. [16] discussed the self-compacting lightweight aggregate concrete under combined compression-shear stress and proposed the damage criteria based on the experimental data. Yu et al. [17] conducted experimental study of plain concrete under combined compression-shear stress. The shear failure stages were identified, and failure characteristics were discussed. Two types of failure criteria which modelled the strength law of plain concrete under the combined stresses were proposed. Hussein and Marzouk [18] carried out four different types of high-strength plate specimens under different biaxial load combinations. The research claimed that the failure modes had no fundamental difference between the high strength concrete and normal concrete. Song et al. [19] analyzed the compression-shear performance of roller compacted concrete and proposed the twin-shear failure criteria based on the test results. Due to the lack of standard of test method for concrete under compression-shear, the specimens from different literature were fabricated variously, which might lead to different conclusions [19,20,21].

The motivation of this research was to study the compression-shear performance of SCC, to compare the performance with normal concrete (NC) and find the characteristics under combines stress, to support future academic research or engineering use of SCC. To achieve the research goal, firstly, in this paper, the combined compression-shear performance experiments as well as the uniaxial compression, uniaxial splitting tensile tests of SCC specimens were carried out by using hydraulic servo machine and material compression-shear testing machine. Then, the shear failure modes of SCC under various compression stress were presented and the stress-strain curve analysis under combined compressive and shear forces are carried out. The characteristics of SCC under combined compressive and shear stresses were extensively analyzed. Finally, based on the experimental data and combing with the results of relevant documents, the corresponding SCC material failure criteria were proposed from the perspective of shear failure strength and octahedral stress space. Comparisons between the experimental and proposed criteria were fully presented.

## 2. Experimental Program

### 2.1. Concrete Mix Proportion

The compressive and shear composite mechanical properties of self-compacting concrete (SCC) are experimentally studied in this paper. The design concrete strength is 35MPa (SCC35) and the self-compacting grade is two. The mix proportion is determined according to China’s Technical Specification for Application of Self-Compacting Concrete (JGJ/T283-2012) [8], as shown in Table 1. The cement used for the concrete material is ordinary Portland cement P.O. 42.5 (P.O. 42.5, Longtan Cement Company, Nanjing, China), and the coarse aggregate is natural stone with particle size ranging from 5 mm to 20 mm, where the parameters are shown in Table 2. Fine aggregate is natural river sand with the maximum particle size of 5mm with the fineness module 2.45. Naphthalene sulfonic acid formaldehyde condensate water reducing agent is adopted, and the dosage of the water reducing agent is 1.0% of the cement mass.

### 2.2. Loading Cases

In order to study the stress state of SCC under combined compression and shear, three different loading modes are designed, which are uniaxial compression, uniaxial splitting tension, and combined compression-shear state. The combined compression-shear experiments include six different axial pressures, namely, 0 MPa, 2 MPa, 4 MPa, 6 MPa, 8 MPa, and 10 MPa, respectively, and the maximum designing axial compression ratio is about 0.30 considering the compression strength of SCC. The loading cases are shown in Table 3. Considering the randomness of concrete materials, three test pieces are adopted for each group of experiments, and the average value of each group is taken for further analysis.

Considering the size limitation of the material compression-shear equipment, and referring some other results in previous literature, the design dimensions of all specimens used in this study are 100 mm × 100 mm × 100 mm, and the size of compression-shear failure surface is 100 mm × 100 mm, correspondingly.

### 2.3. Test Equipment and Loading Schemes

The uniaxial compression and uniaxial splitting tensile tests adopt Reger RE-8060 hydraulic servo machine (Reger instrument, Shenzhen, China). The uniaxial compression tests apply load-controlled method with the loading rate 0.3 MPa/s. The uniaxial splitting tensile tests adopted displacement-controlled loading method with the loading rate 1 mm/min. The loading procedure stopped when the specimen was damaged. SCC compression-shear composite stress test was carried out via CSS-283 material testing machine produced by Changchun Testing Machine Research Institute (Changchun, China), which obtained the ultimate shear load of concrete by direct shear. The machine is equipped with independent load and displacement sensors in both vertical and transverse directions. The equipment consisted of a two-directional loading device controlled by two external digital controllers designed by the Germany company Doli. Each axis of the CSS loading machine has two displacement sensors installed (CD375-5, Changchun Testing Machine Research Institute, Changchun, China). In this study, only the horizontal sensors (CD375-5, Changchun Testing Machine Research Institute, Changchun, China) were utilized. The maximum error of the load sensor is 0.5% of its range (0.5% × 50 t), and the error of the displacement sensor is 5 × 10^−3^ mm whose maximum measuring limit is ± 3 mm which can meet the experimental requirements.

**Figure 1:**
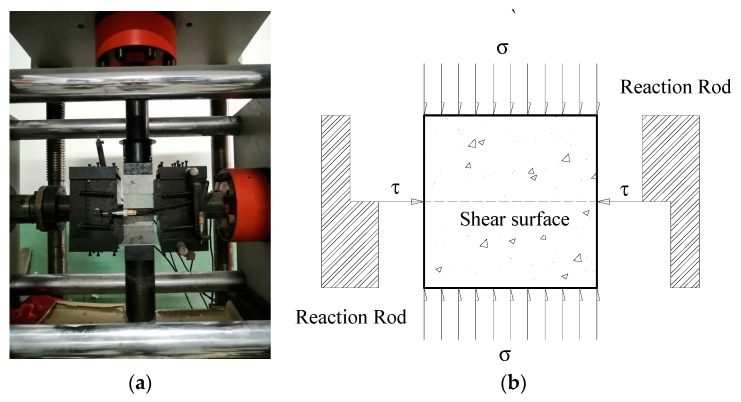
A schematic diagram of compression-shear loading equipment and loading method: (**a**) Loading equipment; (**b**) Sketchy picture of loading method.

The fixed vertical loading method was adopted in the SCC compression-shear composite experiments, i.e., the vertical compression load stayed constant during the increasing of horizontal shear load. The vertical axial loading and the preloading of horizontal direction were completed by force-control method. The experimental procedure was designed as following: firstly, the axial loading was set to the design value in the vertical compression direction and was applied by the vertical pressure head with the rate of 0.5 MPa/min and secondly, in the horizontal direction (shear direction) 0.5 KN load was preloaded for 5 min to stabilize the system, and make sure that the specimen and the horizontal pressure head were tightly attached. Thirdly, the horizontal shear load was applied by displacement-control method, at the loading rate 0.2 mm/min until the failure of specimen. When the lateral load was formally applied after preloading, the horizontal sensors started to collect data. During the compression-shear test, the horizontal load and displacement were recorded simultaneously. Figure 1 is a schematic diagram of compression-shear loading equipment (a) and sketchy loading method (b).

## 3. Experimental Results and Analysis

### 3.1. Shear Failure Modes of SCC

**Figure 2:**
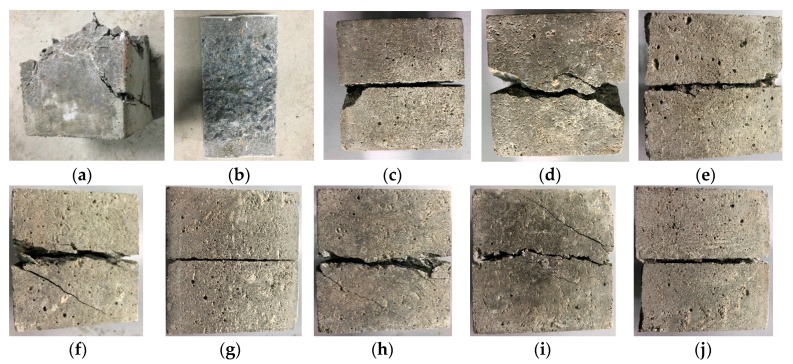
The shear failure modes of different experiments: (**a**) Uniaxial SCC-C; (**b**) Uniaxial splitting tensile SCC-T; (**c**) Shear failure surface SCC-CS-0; (**d**) Lateral shear failure surface SCC-CS-0; (**e**) Shear failure surface SCC-CS-2; (**f**) Lateral shear failure surface SCC-CS-2; (**g**) Shear failure surface SCC-CS-6; (**h**) Lateral shear failure surface SCC-CS-6; (**i**) Shear failure surface SCC-CS-10; (**j**) Lateral shear failure surface SCC-CS-1.

The different shear failure modes of SCC under uniaxial compression, uniaxial splitting tensile and compression-shear experiments are obtained by using hydraulic servo testing machine and material compression shear testing machine, as shown in Figure 2. Shear failure modes in this manuscript refer to not only the pure shear modes, also the ones under different axial compression stress.

The failure mechanism of SCC under uniaxial compression is the same as that of normal concrete (NC). Under axial load and Poisson effect, tensile strain is formed in the horizontal direction perpendicular to the axial direction. With the increase of axial load, when the horizontal strain of the specimen exceeds the ultimate tensile strain, cracks appear inside the specimen and the concrete gradually breaks down, as shown in Figure 2a. The failure mode of SCC under uniaxial compression is oblique shear failure, which is different from that of NC. The uniaxial compression failure mode of NC usually forms an "octagonal" quadrangular pyramid form with connections on both sides. The most serious failure part is located at the middle of the test piece. The upper and lower loading ends are seldom damaged due to the constraint of steel plates, while SCC presents inclined cracks from top to bottom, and the upper and lower ends of the test piece are also damaged. This is due to the fact the SCC has less coarse aggregate and higher material compactness. The test results in this paper are consistent with the failure mode of self-compacting lightweight aggregate concrete reported by Z. Yu et al. [20,22]. The splitting tensile failure mode of SCC is shown in Figure 2b. Under splitting tensile load, the specimen is damaged when the tensile strain reaches the ultimate tensile strain of the material, causing the failure of cement gel layer and aggregates on the splitting surface. This is different from the failure mode of NC, because NC often uses coarse aggregate with higher strength, which is not easy to damage. However, SCC has more fine aggregate and cementing material with higher proportion of bonding stress, which will make the aggregates break at the time of splitting failure.

As one can see from the external shape of the shear failure surface, the SCC compression-shear failure modes are similar under different axial loads, as shown in Figure 2c–j. An obvious failure surface appears after the composite stress experiment is completed, but the failure modes on two sides of the shear surface are different: the failure surficial line at the loading side is relatively flat, while the one on the supporting side is rather tortuous, accompanied by oblique shear cracks. In general, with the increase of axial load, the width of the cracks gradually decreases.

**Figure 3:**
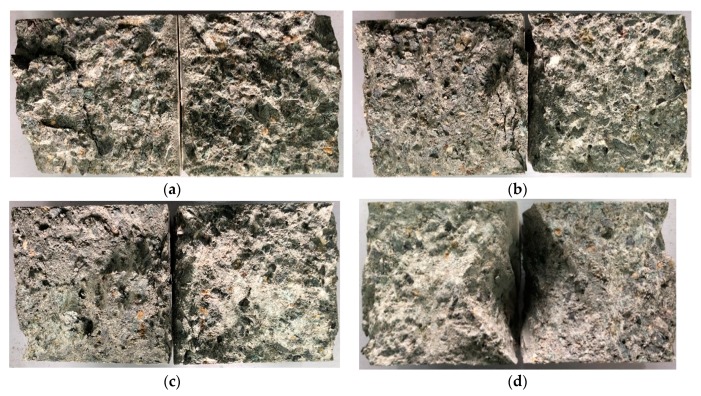
The internal shear failure surface of compression-shear experiments: (**a**) Shear failure surface of SCC-CS-0; (**b**) Shear failure surface of SCC-CS-2; (**c**) Shear failure surface of SCC-CS-6; (**d**) Shear failure surface of SCC-CS-10.

Meanwhile, as one can see from the internal situation of shear failure surface, when the axial pressure is low (0–4 MPa), under the shear load shown in Figure 3a,b, the failure surface is rough, and more visible irregular cracks are generated inside the concrete, and small pieces of concrete flake off. However, with the increase of axial pressure (6–10 MPa), as shown in Figure 3c,d, the spalling of the failure surface decreases, at the same time, there are no visible cracks inside the specimen, while the concrete gradually presents obvious semicircular and splayed compression failure characteristics. The compression-shear failure feature of SCC is similar with the report of Song et al. [19]. When the axial pressure increases, the shear surface becomes coarser and the compressive failure feature is more significant.

### 3.2. Uniaxial Compression and Tensile Strength of SCC

**Figure 4:**
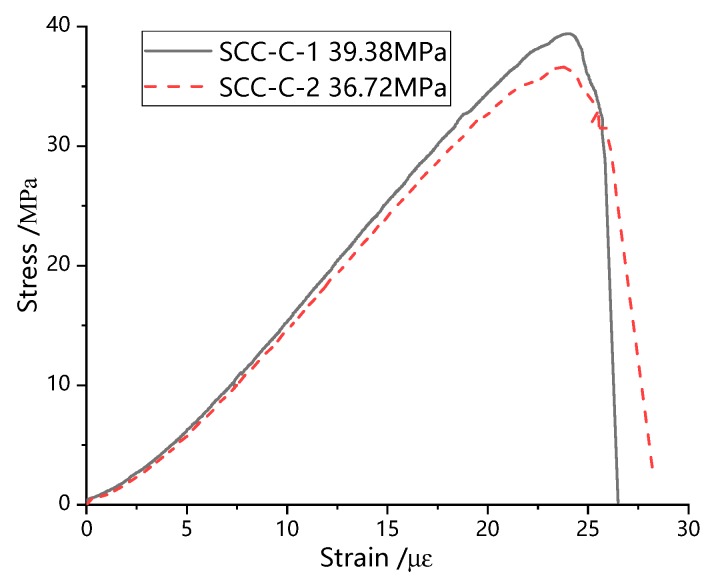
Uniaxial compression stress-strain curve of SCC.

**Figure 5:**
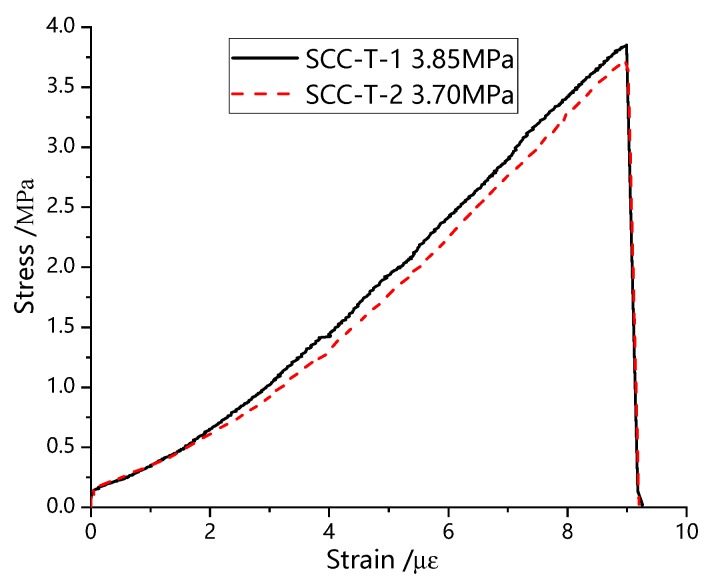
Uniaxial split tensile stress-strain curve of SCC.

According to the Chinese provisions of Standard for Test Method of Mechanical Properties On Ordinary Concrete (GB50081-2002) [23], the typical curves of uniaxial compression and uniaxial splitting tensile experiments of SCC are obtained, as shown in Figure 4 and Figure 5.

(1) $$f_{t}=\frac{2F}{\pi A}$$

where $f_{t}$ is the splitting tensile strength of concrete, *F* the splitting failure load, and *A* the splitting surface area of concrete specimens (in this paper 100 mm × 100 mm).

As can be seen from Figure 4, the development trend of stress-strain curve of SCC under uniaxial compression can be divided into two parts: elastic ascending section, and descending section. The stress-strain curve is smoothing and continuous, and the descending rate is significantly higher than the ascending section, showing obvious brittle failure characteristics and has little plastic deformation. The uniaxial splitting tensile stress strain curve of SCC is shown in Figure 5. Compared with the stress-strain curve under compression, the brittle failure characteristic of SCC under uniaxial splitting tension is more obvious.

By analyzing the test results of uniaxial compression and uniaxial splitting tensile specimens, the axial compressive strength $f_{cu}=36.15MPa$ and uniaxial splitting tensile strength $f_{tu}=3.21MPa$ of SCC are obtained.

### 3.3. Stress-Strain Curve under Combined Stress of Compression and Shear

**Figure 6:**
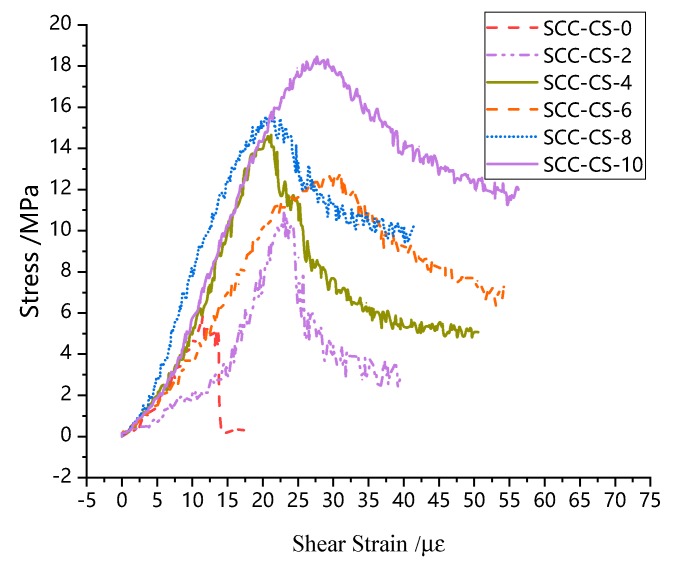
Stress-strain curves under different pressures.

**Figure 7:**
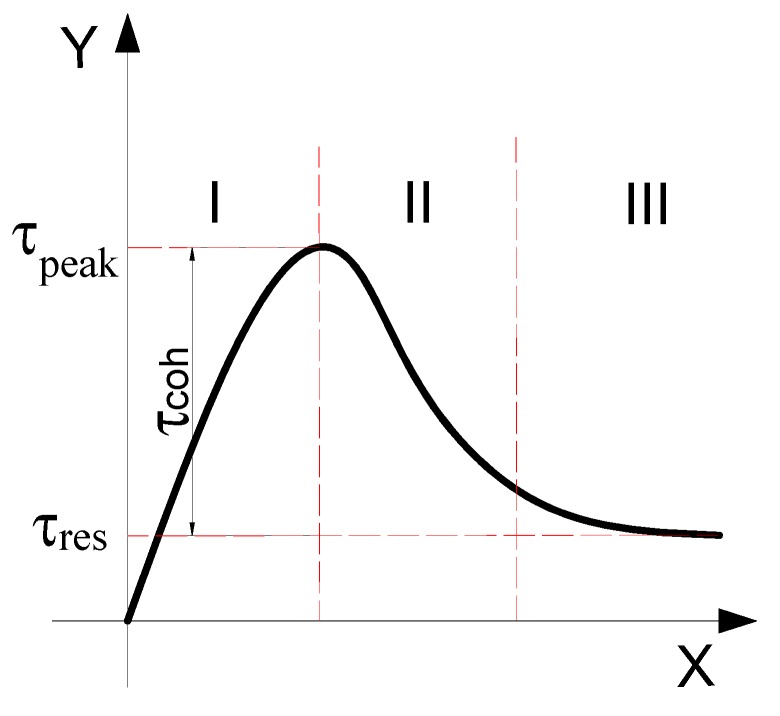
Division of stress-shear stress-strain curves at different stages.

Figure 6 is the part of stress-strain curves from SCC composite compression-shear tests. From the shape of the curves, it can be seen that the stress-strain curve under the combined action of SCC compression and shear can be divided into three parts: rising stage I, falling stage II, and stationary stage III, as shown in Figure 7, which is completely different from the curve of SCC under uniaxial compression or uniaxial splitting tensile test as shown in Figure 4 and Figure 5. Meanwhile both the rising section I and the falling section II have obvious nonlinear characteristics. The resistance of the stationary section III is nearly constant, and the slope of the curve tends to be zero, which is similar with the concrete spalling stage reported in literature [17] when the concrete specimen shows almost constant resistance.

According to recent researches [24,25], the shear strength of concrete $\tau_{peak}$ under combined compression and shear can be divided into three parts, i.e., aggregate interlocking force $\tau_{ui}$, interfacial friction force $\tau_{uf}$, and cohesive stress $\tau_{uc}$, which have the following relationship:

(2) $$\tau_{peak}=\tau_{ui}+\tau_{uf}+\tau_{uc}$$

However, it is difficult to separate aggregate interlocking stress $\tau_{ui}$ and interfacial friction stress $\tau_{uf}$ completely because they are tangled with each other and change with compressive load. After SCC specimens enter stage III, the rate of the stress-strain curve will tend towards zero. At this time, the relative deformation of the shear surface of concrete is relatively large. This study assumes that $\tau_{uc}$ and $\tau_{ui}$ have disappeared at this time, and the residual stress is provided by $\tau_{uf}$ only. Similar to the method in [19], the former two effects are added together, and the peak shear strength is written into the sum of two terms:

(3) $$\tau_{peak}=\tau_{coh}+\tau_{uf}=\tau_{coh}+\tau_{res}$$

where $\tau_{coh}=\tau_{ui}+\tau_{uc}$ is the generalized cohesive stress and $\tau_{res}$ is the residual friction stress at stage III. Then the generalized cohesive stress can be subtracted from the peak shear stress after the residual stress is obtained from the test curve. Columns 2 to 7 in Table 4 show the test results of different strengths obtained through compression-shear tests.

Through SCC compression-shear tests, the shear strength of the material under different axial forces can be obtained, the tested and average values are shown in columns 2 and 3 of Table 4. Then, the relationship between shear strength and residual stress, namely Equation (3), can be applied to calculate the cohesive stress, as shown in columns 4 and 5 of Table 4.

### 3.4. Shear Strength under Different Axial Loading

**Figure 8:**
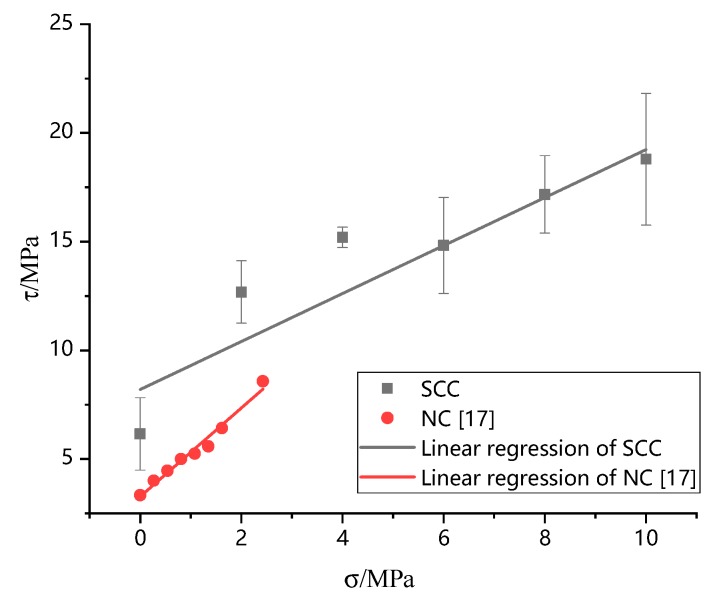
Relationship between axial pressure and peak shear strength.

The relationship between shear strength and axial pressure of concrete under compressive shear load can be described by Mohr–Coulomb model [17,26,27], i.e.,:

(4) $$\tau_{peak}=\mu \sigma +c$$

where μ,c are the coefficient of frictional and cohesive stress, and $\tau_{peak},\sigma$ are shear strength and axial pressure, respectively. According to the test results in Table 4, the peak stress and axial pressure of SCC are fitted linearly, and the results are shown in the first row of Table 5. Figure 8 shows the relationship between axial pressure and shear strength in SCC compression-shear tests. To figure out the characteristics properties of SCC more clearly, the compression-shear results of normal concrete by Yu et al. (2018) are also included in the figure for comparing purpose.

Figure 8 shows the experimental fitting results of the pressure and shear strength of NC in [17]. As can be seen from the first and third rows in Table 5 and Figure 8, the friction coefficient *µ* of SCC is obviously smaller (about 44% smaller) and the bond stress is much larger (about 158% bigger) than those of NC. The phenomenon should be caused by the instinct properties of SCC for the content of fine aggregate in SCC leads to denser internal cement stone structure compared with NC. At the same time, the coarse aggregates in NC would cause larger friction stress for the interlocking effect was more obvious than in SCC. These two factors contributed to the difference of friction coefficient and cohesive stress. In the design of structures or structural members, in the shear failure stage, the results of this research indicated that SCC could not provide the same friction resistance as NC. It is unsafe to design SCC member following the instructions of NC in shear failure mode. It should be pointed out that the axial compression ratio in this paper was different from those in references [16,17], and the mechanical properties of SCC under the condition of large axial compression ratio need further experimental verification.

### 3.5. Residual and Cohesive Stress under Different Axial Loading

**Figure 9:**
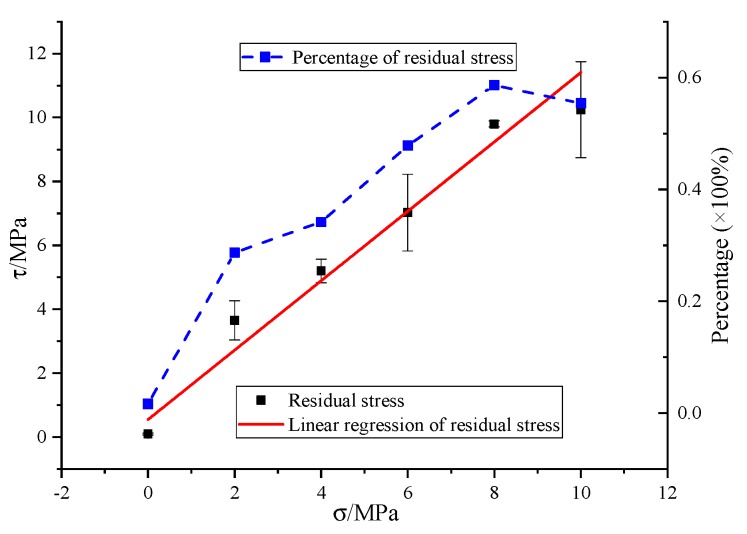
Relationship between residual stress and axial pressure.

Based on the data obtained from SCC compression-shear tests, the relationship between residual stress and axial pressure was mathematically fitted, shown in Figure 9. There exists a good linear relationship between residual stress and axial pressure, which can also be described by the Mohr–Coulomb relation of Equation (4). The friction coefficient and cohesive stress of the fitting curve are shown in second row of Table 5.

Comparing the shear strength and the fitting results of residual strength and axial stress in Table 5, it can be found that the friction coefficient *µ* changes little while the cohesive stress *c* decreases greatly, which indicates that the proportion of bonding stress in residual stress is very small which verifies the rationality in Equation (3) that we put cohesive stress $\tau_{uc}$ away from residual stress $\tau_{res}$, and that the SCC compressive shear strength divided by Equation (3) in this paper is reasonable. Figure 9 also shows the ratio of residual stress to shear strength under different axial pressures. With the increase of axial pressure, the proportion of residual stress in shear strength gradually increases, with the maximum proportion of 58.6% at 8 MPa and a slight decrease at 10 MPa.

**Figure 10:**
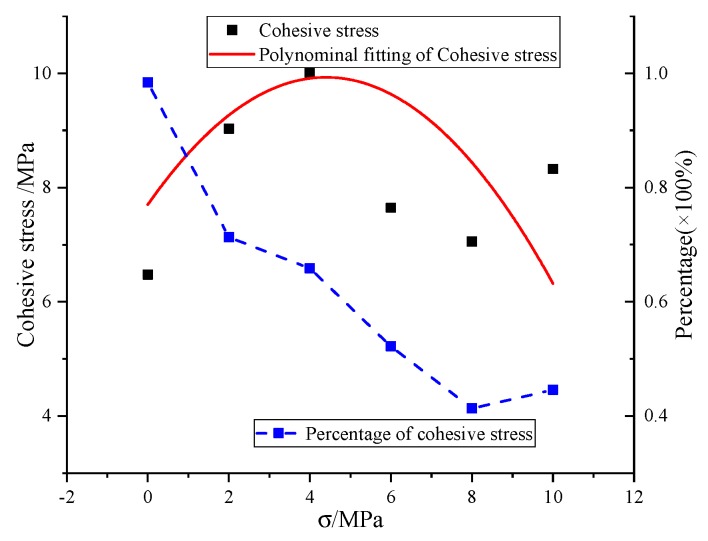
Relationship between cohesive stress and axial pressure.

The cohesive stresses of SCC concrete under different axial pressures are plotted in Figure 10. It can be found that the cohesive stress increases first and then decreases with the increase of axial pressure in a limited boundary, i.e., between 6 MPa and 10 MPa, which was also observed by Bresler et al. [28] and Deng et al. [25]. The cohesive stress obtained by the linear relationship between shear strength and axial pressure in Table 5 is the average value.

Figure 10 also shows the ratio of cohesive stress to shear strength. It can be observed that the proportion of cohesive stress in shear strength gradually decreases and tends to be stable with the increase of axial pressure. When the axial compression stress is 8 MPa, the minimum proportion is 41.4%, and the average value of cohesive stress is 8.46 MPa. Deng et al. [25] calculated the ratio of bond stress of ordinary concrete and roller compacted recycled concrete to shear strength. The results show that the cohesive strength between ordinary concrete and recycled concrete accounts for 10%–30% of the shear strength, while this study found that the ratio of SCC cohesive strength to shear strength is about 41.3%–98.4%, which is significantly higher than that of ordinary concrete or roller compacted recycled concrete. This depends on the fact that SCC has less coarse aggregate and the cohesive stress is mainly caused by the interaction between fine aggregate and binder, which determines that the cohesive strength of SCC takes a significantly higher proportion of shear strength.

## 4. Failure Criteria of SCC

### 4.1. Failure Criteria of Octahedral Space Stress

Under the combined action of compression and shear, the principal stress of SCC specimens can be written in the following form:

(5) $$\left\{ \begin{array}{l} \sigma_{1}=\frac{\sigma }{2}+\frac{\sqrt{\sigma^{2}+4\tau^{2}}}{2}\\ \sigma_{2}=0\\ \sigma_{3}=\frac{\sigma }{2}-\frac{\sqrt{\sigma^{2}+4\tau^{2}}}{2} \end{array}\right.$$

where $\sigma_{1},\;\sigma_{2},\;\sigma_{3}$ are the first, second, and third principal stresses, σ, τ are axial stress and shear stress, respectively, then the corresponding eight-flour stress can be written as:

(6) $$\begin{array}{l} \sigma_{oct}=\frac{1}{3}\left(\sigma_{1}+\sigma_{2}+\sigma_{3}\right)=\frac{\sigma }{3}\\ \tau_{oct}=\frac{1}{3}\sqrt{{\left(\sigma_{1}-\sigma_{2}\right)}^{2}+{\left(\sigma_{2}-\sigma_{3}\right)}^{2}+{\left(\sigma_{3}-\sigma_{1}\right)}^{2}}=\frac{1}{3}\sqrt{2(\sigma^{2}+3\tau^{2})} \end{array}$$

where $\sigma_{oct},\tau_{oct}$ are normal and shear stress of octagonal element.

According to the relevant research on concrete strength criteria under composite load, the strength criteria of different concrete can be expressed in three basic forms [29,30], among which the multi-parameter strength criteria proposed by Willam et al. [31] and Kang [32] can be expressed in the form of the following Equation (7):

(7) $$\frac{\tau_{oct}}{f_{c}}=A+B\frac{\sigma_{oct}}{f_{c}}+C{\left(\frac{\sigma_{oct}}{f_{c}}\right)}^{2}$$

where $f_{c}$ is the uniaxial compressive strength of concrete, A,B,C are the coefficients related to material, which can be determined by mathematical regression.

**Figure 11:**
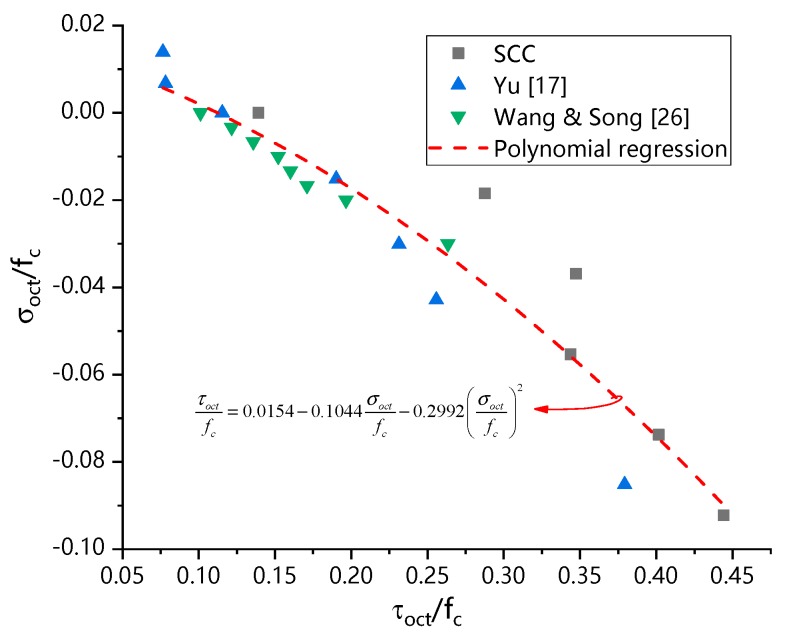
SCC failure criterion of octahedral stress space.

Similarly, using the measured data in this paper and the relevant data in literature [17,33], the strength criterion of spatial stress of the octahedral bodies was set up, and the results were shown in Equation (8) and Figure 11. The difference between the fitted value and the measured value was small, which indicated that the SCC compression-shear composite stress state was well fitted by using the failure criterion of octahedral stress space as shown in Equation (8):

(8) $$\frac{\tau_{oct}}{f_{c}}=0.0154-0.1044\frac{\sigma_{oct}}{f_{c}}-0.2992{\left(\frac{\sigma_{oct}}{f_{c}}\right)}^{2}\;R^{2}=0.914$$

Although the data in references [17,33] were not obtained from SCC but from recycled concrete and plain normal concrete, they were collected from the same kind of experiments under similar loading conditions. Furthermore, the criteria of octahedral stress space were independent with the compressive strength of concrete and within the scope of principle stress scope, it was reasonable to derive the failure criteria from different kinds of concrete. The degree of coincidence of the fitting curve showed clear agreement with the experimental results.

### 4.2. Failure Criteria Based on Unified Twin Shear Strength Theory

Due to the large difference in tensile and compressive strength of concrete materials, different researchers have proposed many yield criteria. The classical Mohr–Coulomb theory considers the influence of shear stress and normal stress, improves the maximum shear stress theory, but cannot consider the influence of the second principal stress. Yu [29] proposed a unified twin shear strength theory based on principal stress space, which can better consider the influence of intermediate principal stress and is suitable for brittle materials such as concrete. From Equation (5) of the principal stress, the unified shear stress criterion can be obtained as follows:

(9) $$f=\sigma_{1}-\frac{1}{1+b}(b\sigma_{2}+\sigma_{3})=\sigma_{s}\;\mathrm{when}\;\sigma_{2}\leq \frac{1}{2}(\sigma_{1}+\sigma_{3})$$

(10) $$f’=-\sigma_{1}+\frac{1}{1+b}(\sigma_{1}+b\sigma_{2})=\sigma_{s}\;\mathrm{when}\;\sigma_{2}\geq \frac{1}{2}(\sigma_{1}+\sigma_{3})$$

Substituting the principal stress Equation (5) into Equations (9) and (10), one obtains:

(11) $$f=\frac{2+b}{2+2b}\sqrt{\sigma^{2}+4\tau^{2}}+\frac{b}{2+2b}\sigma =\sigma_{s}\;\mathrm{when}\;\sigma \geq 0$$

(12) $$f=\frac{2+b}{2+2b}\sqrt{\sigma^{2}+4\tau^{2}}-\frac{b}{2+2b}\sigma =\sigma_{s}\;\mathrm{when}\;\sigma \leq 0$$

Yu [5] pointed out that different values of b in Equations (11) and (12) will lead to different yield criteria. If b = 0, the yield criterion of single shear stress can be obtained, and if b = 1, the yield criterion of double shear stress can be obtained. When b = ∞ the maximum strength criterion can be obtained. If the influence of intermediate principal stress is taken into account, Equations (11) and (12) can be rewritten as follows:

(13) $$f=(1+3\beta )\sigma +(3+\beta )\sqrt{\sigma^{2}+4\tau^{2}}=4C\;\mathrm{when}\;\sigma +\beta \sqrt{\sigma^{2}+4\tau^{2}}\geq 0$$

(14) $$f=(-1+3\beta )\sigma +(3-\beta )\sqrt{\sigma^{2}+4\tau^{2}}=4C\;\mathrm{when}\;\sigma +\beta \sqrt{\sigma^{2}+4\tau^{2}}\leq 0$$

where β is the coefficient of influence of concrete intermediate principal stress and *C* is the concrete strength parameter.

**Figure 12:**
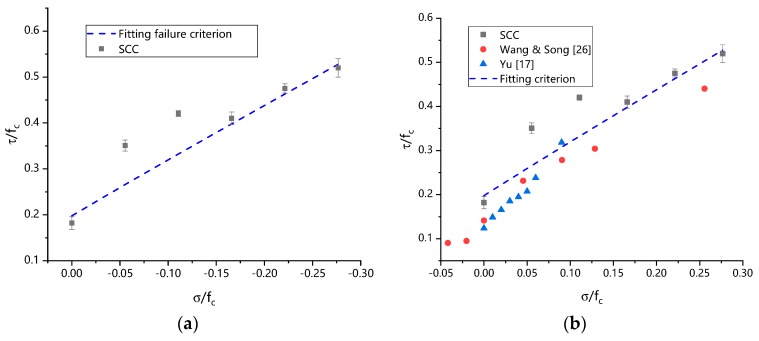
The unified twin shear failure criterion of SCC: (**a**) Failure criterion of twin shear strength theory; (**b**) Comparison of the proposed failure criterion with multiple experimental data.

Using shear strength values and unified strength theoretical Equations (13) and (14) obtained from different axial force tests, the comparison between unified failure criteria and test data under combined compression and shear of SCC concrete is shown in Figure 12a. The two parameters of the unified failure criterion obtained from fitting and inversion calculation of test data are β=13.49, C=43.10, respectively. As can be seen from Figure 12a, the consistency between the strength criterion obtained by the unified strength criterion and the test data agrees with each other quite well, which indicates that the unified double shear strength criterion can effectively simulate the stress state of SCC concrete under composite compression and shear. Furthermore, to validate the proposed failure criterion, the experimental data of concrete under combined compression and shear status from other literatures, such as Yu [17] and Wang [33], are also included in Figure 12b. It clearly can be seen that the proposed failure criterion can also represent the ultimate stress conditions, indicating that the unified twin shear failure criterion could be applied to the concrete under combined compression and shear.

## 5. Discussion

In this section, two critical key points are analyzed and discussed: (1) The failure mechanism and failure modes of SCC under compression-shear stress; (2) the reason that different kinds of concrete are included in the derivation of failure criteria.

The failure mechanism of SCC under combined compression-shear stresses is the same in different concretes. The composite compression-shear strength failure mode of SCC under high axial compression is similar with NC [17], which tends to be crashed by axial compressive load. When the compressive stress is applied on the specimen, the shear strength of the concrete will increase for the friction stress and the cohesive stress between cement and aggregate would both increases. The different failure appearances of SCC and NC may be contributed to the following two facts. First, the shear failure is due to the loss of cohesive stresses under compression-shear experiments and the percentage of cohesive stress takes a much larger share than the one of NC. Second, the residual stress after shear failure of SCC is mainly composed of the friction stress which depends on the axial comprehensive stress. The friction factor of SCC is much smaller than NC for the lack of interlocking effect of coarse aggregates. These two facts are contributed to the percentages of fine and coarse aggregates in SCC which are totally different from NC. Although in current study, the shear strength of SCC increases with axial compression, the shear performance under higher axial pressure needs further experiments and attention.

Researches on failure criteria of concrete have been extensively carried out and led to unified strength or failure laws despite of the types of concrete [30]. Firstly, SCC is a special kind of concrete with different dosage of aggregates, thus the macro mechanical performance should agree with normal concrete if the factor of compression strength is excluded, which had been verified by Khayat et al. [12]. On the other side, the experimental results of combined compression-shear stress of SCC are relatively rare and considering the discreteness of concrete properties, it is sensible to choose more existing data to propose or validate the failure criteria. Secondly, loading condition is critical to the performance of shear strength of concrete specimen. So, all the data collected from literature have an identical loading condition, that is, two axial stresses with one compression and one shear, although the types of concrete containing recycled concrete and plain concrete. The regression results show favorable agreement and the proposed criteria have simple mathematical expression which could be easily applied. Still, it should be pointed out that the compression ratio (the compressive stress applied by the pressure head to the compressive strength of SCC) was relatively small, the performance under high compression ratio still needs to be investigated further.

## 6. Conclusions

In this paper, the mechanical performance of SCC under combined compression-shear stress is tested and theoretically analyzed. Firstly, the shear failure modes and stress-strain curves of SCC specimens under different axial pressures were obtained through experiments. Secondly, the shape of the compression-shear test curve of SCC specimens was analyzed, and the characteristic values of the curve were obtained. Thirdly, according to the test results, the characteristics and proportional relationships of cohesive and friction stress (including interlocking stress) in shear strength of SCC were analyzed. Finally, two kinds of failure criteria based on compression-shear strength and octahedral stress space are studied. The following conclusions can be obtained:

1. The failure mode of SCC under uniaxial compression was different from that of NC, which was the inclined shear failure mode. With the increase of axial compression stress, the internal cracks of shear surface in SCC specimens gradually disappear, and the failure mode became the axial compressive failure mode under the combined compressive-shear stress state accordingly.

2. The shear strength of SCC under combined compression-shear stress can be divided into two parts: general cohesive stress and residual friction stress. The relationship between the residual strength, shear strength and axial pressure can be predicted by Mohr-Coulomb relationship.

3. The friction coefficient of shear strength in SCC was much smaller than that in NC, which meant that in the shear failure state, SCC cannot afford enough shear resistance as NC. Meanwhile, the proportion of cohesive stress and residual friction stress of SCC was totally different from NC. The cohesive stress took a larger part in SCC than in NC.

4. Based on SCC’s combined compression-shear stress test data and the related test data in the existing literature, the unified strength failure criterion as well as the failure criteria based on octahedral stress space were proposed. The experimental and regression results demonstrate that the proposed failure criteria can describe the failure laws of SCC properly.

## Figures and Tables

**Table 1:** Concrete mix proportion of self-compacting concrete (SCC) (kg/m^3^).

Concrete Grade	Cement	Water	Coarse Aggregate	Fine Aggregate	Mineral Powder	Water Reducer
SCC35	385	166	310	720	197	3.85

**Table 2:** Basic physical properties of coarse aggregate.

Coarse Aggregate Types	Apparent Density (/kg·m^−3^)	Bulk Density (/kg·m^−3^)	Crushing Index (/%)	Water Absorption Rate (/%)	Particle Size Ranges (/mm)
Nature	2700	1465	9.1	1.2	5–20

**Table 3:** Loading cases of self-compacting concrete.

Index	Loading Cases	Axial Pressure	Index	Loading Condition	Axial Pressure
SCC-C	uniaxial compression	/	SCC-CS-4	composite compression-shear	4 MPa
SCC-T	uniaxial splitting tensile	/	SCC-CS-6	compression-shear composite	6 MPa
SCC-CS-0	composite compression-shear	0 MPa	SCC-CS-8	composite compression-shear	8 MPa
SCC-CS-2	composite compression-shear	2 MPa	SCC-CS-10	composite compression-shear	10 MPa

**Table 4:** SCC compression-shear test results under different axial stress.

Axial Pressure σ/MPa	$\mathrm{Shear}\;\mathrm{Strength}\;\tau_{peak}/\mathrm{MPa}$			$\mathrm{Cohesive}\;\mathrm{Strength}\;\tau_{coh}/\mathrm{MPa}$			$\mathrm{Residual}\;\mathrm{Strength}\;\tau_{res}/\mathrm{MPa}$		
Item (1)	Test data (2)	Average (3)	STD (4)	Test data (5)	Average (6)	STD (7)	Test data (8)	Average (9)	STD (10)
0	5.40/5.01/8.07	6.16	1.67	5.27/5.29/8.87	6.48	1.66	0.12/0.06/0.09	0.09	0.03
−2	13.59/11.03/13.43	12.68	1.43	9.32/7.99/9.79	9.03	0.93	4.27/3.04/3.64	3.65	0.62
−4	15.71/15.10/14.79	15.20	0.47	10.09/10.16/9.76	10.00	0.21	5.62/4.94/5.03	5.20	0.37
−6	12.50/16.90/15.07	14.82	2.21	6.43/8.61/7.90	7.65	1.06	5.90/8.29/6.88	7.02	1.20
−8	18.93/17.21/15.37	17.17	1.78	9.22/6.61/5.33	7.05	1.83	9.71/9.90/9.80	9.80	0.10
−10	22.03/18.32/16.02	18.79	3.03	10.66/7.11/7.21	8.33	1.92	11.37/10.83/8.81	10.34	0.38

**Table 5:** Relationship between shear strength, residual strength, and axial force.

Item (1)	Friction Coefficient *µ* (2)	Cohesive Stress *c* (3)	*R*^2^ (4)
SCC Shear strength	1.1065	8.4566	0.932
SCC Residual strength	1.0866	0.5447	0.989
NC Shear strength [17]	1.96	3.27	0.972

## Data Availability

The datasets generated during and/or analyzed during the current study are available from the corresponding author on reasonable request. The datasets in references are clearly listed and could be accessed according to the published references.
